# Automatic hyoid bone detection in fluoroscopic images using deep learning

**DOI:** 10.1038/s41598-018-30182-6

**Published:** 2018-08-17

**Authors:** Zhenwei Zhang, James L. Coyle, Ervin Sejdić

**Affiliations:** 1 Department of Electrical and Computer Engineering, Swanson School of Engineering, University of Pittsburgh, Pittsburgh, PA 15261 USA; 2 Department of Communication Science and Disorders, University of Pittsburgh, Pittsburgh, PA 15260 USA

## Abstract

The displacement of the hyoid bone is one of the key components evaluated in the swallow study, as its motion during swallowing is related to overall swallowing integrity. In daily research settings, experts visually detect the hyoid bone in the video frames and manually plot hyoid bone position frame by frame. This study aims to develop an automatic method to localize the location of the hyoid bone in the video sequence. To automatically detect the location of the hyoid bone in a frame, we proposed a single shot multibox detector, a deep convolutional neural network, which is employed to detect and classify the location of the hyoid bone. We also evaluated the performance of two other state-of-art detection methods for comparison. The experimental results clearly showed that the single shot multibox detector can detect the hyoid bone with an average precision of 89.14% and outperform other auto-detection algorithms. We conclude that this automatic hyoid bone tracking system is accurate enough to be widely applied as a pre-processing step for image processing in dysphagia research, as well as a promising development that may be useful in the diagnosis of dysphagia.

## Introduction

Dysphagia, a common condition among older individuals, is defined as an impairment in swallowing function during eating and drinking^1^. Dysphagia causes subjective discomfort and objective difficulty in the formation or transportation of a bolus from mouth to stomach, and prevention of errant entry of swallowed material into the airway. Dysphagia is a frequent clinical sign in patients with stroke, head and neck cancer and a variety of other medical conditions^2–4^. The prevalence of dysphagia is very high: stroke is the most commonly reported etiology with over 50% of patients exhibiting dysphagia in the immediate post-onset stage of recovery, diminishing to a lower prevalence of around 11% within 6 months of onset^5^. Additionally, chronic dysphagia affects 7.2% of people with other neurological diseases, and 4.9% of patients treated for head and neck cancer^6^. Up to 40% of people over 65 years old and more than 60% of adults in nursing home^7^ suffer from dysphagia. It is estimated that 25–50% of Americans over 60^2^ and 17% of citizens over 65 in Europe^8^ will suffer from dysphagia, leading to increased risk of poor nutrition or dehydration. The variation in estimation may be due to different definitions of dysphagia, the method of swallowing assessment and the number of patients investigated. As a more immediate clinical consequence, dysphagia may lead to misdirection of food and colonized saliva into the airway, possibly causing pneumonia and chronic lung disease. In many cases aspiration occurs without any obvious clinical signs of dysphagia (silent aspiration), postponing early identification and preventive treatment therefore lowering patient survival^9^. Efforts to accurately evaluate swallowing function early after the onset of conditions leading to dysphagia can mitigate many of these health risks^10^.

The videofluoroscopic swallowing studies (VFSS), also known as modified barium swallow study, is the gold standard test for dysphagia evaluation^11–14^. VFSS, unlike bedside clinical examination, enables the examiner to visualize oral, pharyngeal and upper esophageal structure and function during patient swallowing. VFSS also evaluate errors of biomechanical coordination that lead to bolus misdirection. Patients with dysphagia may not exhibit overt signs of swallowing problems at the bedside. VFSS excels at allowing clinicians to identify occult disorders in airway protection and biomechanical errors leading to impaired airway protection and transfer of food to the digestive system. Airway closure and upper esophageal sphincter opening are largely influenced by the timing and displacement of the hyolaryngeal complex during the pharyngeal stage of swallowing. During VFSS, the hyoid bone is the most salient anatomic structure for detecting hyolaryngeal motion^15^. Hyolaryngeal excursion is an important feature considered by clinicians and researchers because disordered motion may signify dysphagia. Clinicians make subjective judgments about the completeness of hyoid displacement by gross visual inspection of VFSS images. In dysphagia research labs, expert judges annotate hyoid position and its key components in each image frame. However, the subjective clinical process is prone to judgment error, and frame-by-frame annotation done by researchers is time consuming and is prone to inter- and intra-rater variation^16^.

Efforts by researchers to develop hyoid tracking methods that combine human judgment with automated image processing and machine learning are still quite limited. Patrick *et al*. proposed a method to define the hyoid bone in a calibration frame by identifying a region of interest manually and using Sobel edge detection to track the hyoid bone in subsequent frames^17^. Hoaasin *et al*. proposed a semi-automatic hyoid bone tracking system that can match the hyoid bone by Haar classifier matching. However, their method still requires manual identification of regions that clearly contain the hyoid bone^18^. Lee *et al*. developed a software platform that extracted the trajectory of the moving hyoid bone by calculating local binary patterns and multi-scale local binary patterns^19^. Kim *et al*. developed software which can track, smooth and segment the hyoid bone motion from VFSS^20^.

Remarkable progress has been made in medical imaging techniques due to the large number of databases and deep convolutional neural networks (CNNs)^21,22^. Currently, the ideas of CNNs are mainly employed in various medical imaging modalities such as conventional X-ray fluoroscopy, MRI and CT for classification and segmentation^23–26^. The medical applications of CNNs techniques are to help clinicians diagnose and classify diseases more quickly, including segmentation of various tissues such as brain and organs; classification of cancer, fractures, neurological diseases and biomedical image retrieval systems. Research based on segmentation and object detection has closely followed the development of CNNs in the last few years. Almost all recent works for the object detectors and segmentation are based on CNNs, a deep architecture using pretraining on ImageNet which is trainable end-to-end. Girshick *et al*. first described Region-based Convolutional Neural Networks (RCNN) that dramatically increased the performance of object detection compared to traditional features based classifiers^27^. Traditional methods usually use sliding windows for region proposal, histograms of gradient orientation (HoG) or scale-invariant feature transform (SIFT) as feature extraction^28,29^, and support vector machine (SVM) and Boosting methods as classifiers^30,31^. Fast-RCNN extended the idea of RCNN and improved system performance by sharing the computation across the proposed image regions^32^. Then, Faster-RCNN improved the region proposer method and sped up the overall process^33^. In this method, only one CNN is trained and the region proposal reused the results of the same CNN instead of running a separate searching algorithm in the previous work. You Only Look Once (YOLO)^34^ and Single Shot MultiBox Detector (SSD)^35^ are existing methods that focus on better computation speed and performance. These two methods classify and regress a set of anchor boxes without using the idea of Regions of Interests. YOLO applies a simpler network structure, predicting bounding boxes and class probabilities directly from the last convolutional feature maps. SSD uses features from different layers progressively to predict the various size of bounding boxes. Features from the early layers were applied to predict the small-sized boxes while features from the latter layers are applied for larger boxes.

In previous research related to the hyoid bone motion, users manually marked a region of interest in the first frame after which their algorithm tracked or detected the motion of hyoid bone. The number of images used in these studies was not representative of a patient population. The hyoid bone motion analysis provides meaningful solutions in clinical research settings. However, the manual tracking is time consuming and impractical in real-life cases. Improved hyoid bone localization and an automatic hyoid bone tracking system can help clinicians provide a quicker assessment of the patient. Therefore, we sought to develop a software platform that can localize the region of interest containing the hyoid bone in subsequent video frames. The proposed method relies on the CNN based object detection method. We hypothesized that our detection algorithms would accurately detect the location of the hyoid bone in each video frame with high accuracy when compared to the gold-standard manual detection method (visual inspection with frame-by-frame plotting).

The paper is organized as follows. Section 2 reports the background and the current state-of-the-art object detection methods; section 3 proposes the methodology, followed by the analysis of the experimental results and discussion; and section 4 concludes the paper.

## Material and Methods

### Data Collection

**Figure 1:**
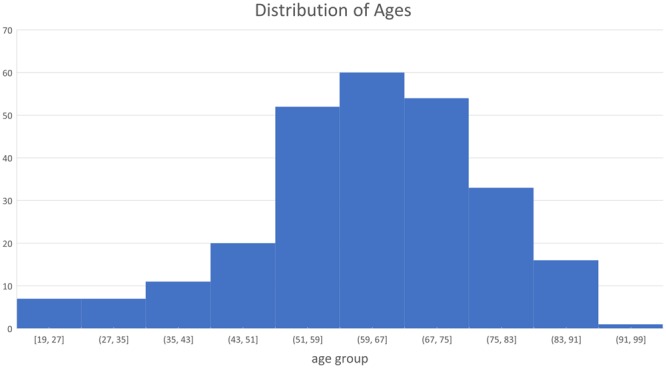
The age range of participants are from 19 to 94. Most of subjects are in the age range 43–83 years old.

In this investigation, 265 patients with swallowing difficulty underwent videofluoroscopic examination at the Presbyterian University Hospital of the University of Pittsburgh Medical Center (Pittsburgh, Pennsylvania). The protocol for this study was approved by the Institutional Review Board at the University of Pittsburgh and all participants provided informed consent. All experiments were performed in accordance with relevant guidelines and regulations. The age range of these subjects was from 19 to 94, and the average age was 64.833 ± 13.56 years old. The distribution of ages is illustrated in Fig. 1. There were no significant differences in hyoid bones between younger and older patients in the detection task. The main difference in the anatomy of the hyoid bone across the lifespan is density and when the greater cornua fuses to the body of the hyoid. Hyoid bone tracking with VFSS relies on identification of landmarks on the body of the hyoid bone without regard to cornua^36^. Patients swallowed radiopaque liquid boluses of different consistencies and volumes as well as pureed food and cookies during their VFSS examination. The volumes and viscosity of material administered to patients were determined during the examinations in real time by clinicians based on factors such as the patient’s history and clinical indications. These liquids included thin liquid (Varibar Thin Liquid with <5 cPs viscosity), and nectar-thick liquid (Varibar Nectar with about 300 cPs viscosity). The position of patients during swallowing was primarily neutral head position though some swallows were performed in a head-neck flexion position. Patients swallowed liquid boluses from a spoon containing 3–5 mL volumes, or self-administered boluses from a cup containing patient self-selected, comfortable volumes between 10–20 mL.

Videofluoroscopy was set at 30 pulses per second (full motion) and video images were acquired at 60 frames per second by a video card (AccuStream Express HD, Foresight Imaging, Chelmsford, MA) and collected into a hard drive with a LabVIEW program. The videos were two-dimensional digital movie clips of 720 × 1080 resolution, and in this investigation, we down-sampled the video clips to 30 frames/second to eliminate duplicated frames.

### Methods

In this investigation, our solution is to build a detection system based on the single shot multibox detector, which is one of the most popular detection algorithms in recent years. The SSD algorithm can generate high detection performance at the cost of high computational complexity. Thus, we also evaluated the performance of several other state-of-the-art detection methods, i.e., Faster-RCNN and YOLOv2, for comparison. The following paragraphs describe the SSD approach, the data set ground truth creation and the training and testing details.

#### Network Architecture

Machine learning has been widely used in medical imaging and videos to help users better understand the properties of these data^37^. Neural networks are one of the most popular types of machine learning models. The basic idea of neural networks is to multiply the input data with layers of weighted connections. Deep neural networks consist of a typical architecture of neural networks, constructed by multiple layers. Each layer implements a series of convolution operators on input, followed by a non-linear activation function, such as a logistic function or a rectified linear unit (Relu). Then a pooling layer is applied to reduce the size of features to the following layers^38^. Popular convolutional neural networks for image tasks include AlexNet^39^, GoogleNet^40^, VGG net^41^ and Residual Net^42^.

The SSD is a feed-forward convolutional neural network built on image classification neural network, called base network, such as VGGNet, ZFNet or ResNet^35^. Eight additional convolutional feature layers are added after these base networks to replace the last few layers of the base networks. The size of these layers decreased progressively and were used as output layers for the prediction of detections at multiple resolutions. SSD integrated both higher and lower feature layers, as the lower layers contain better location information and the higher layers have more image details^43^. The images are divided into different grid sizes which are associated to default bounding boxes. The correspondence between the position of the default box and the feature cell are fixed. SSD predicts the objects based on default boxes instead of predicting the bounding boxes directly. The default boxes are assigned with different scales and aspect ratios, which provides information on different object scales. The scale of each feature map is manually designed as:

$${s}_{k}={s}_{min}+\frac{{s}_{max}-{s}_{min}}{m-1}(k-1),\,\,k\in [1,m]$$

where *m* is the number of feature maps used for prediction. *s*_*min*_ is 0.2 and *s*_*max*_ is 0.9.

**Figure 2:**
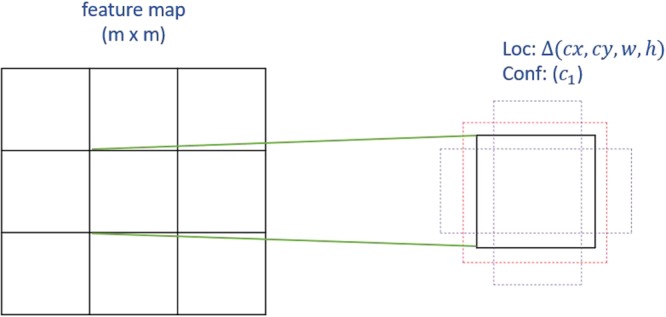
The idea of default boxes applied in SSD. For each default box, the offsets and confidence for categories are predicted.

Each feature map cell is correspondent to 6 default boxes, which are assigned with different aspect ratios, denoted as ${\alpha }_{\gamma }=\{1,2,3,\frac{1}{2},\frac{1}{3}\}$. The width and height of the default box is computed as ${w}_{k}^{\alpha }={s}_{k}\sqrt{{\alpha }_{\gamma }}$ and ${h}_{k}^{\alpha }={s}_{k}/\,\sqrt{{\alpha }_{\gamma }}$. For the aspect ratio of 1, another scale ${s^{\prime} }_{k}=\sqrt{{s}_{k}{s}_{k+1}}$ is added for the default box as well. The center of each default box is set at $(\frac{i+0.5}{|{f}_{k}|},\frac{j+0.5}{|{f}_{k}|})$, and $|{f}_{k}|$ is the size of k-th feature map. By using these default boxes with various scales and aspect ratios from all locations of added feature maps, SSD predictions can cover different input sizes and shapes. Figure 2 illustrates the idea of default boxes.

A set of convolutional filters are applied to the added features layers to perform the bounding box regression and category classification. For each feature layer of size *m* × *n* with *p* channels, a 3 × 3 × *p* small kernel filter is applied to produce one value at each feature map cell, where the outputs are classification scores as well as the offsets relative to the bounding box shape.

**Figure 3:**
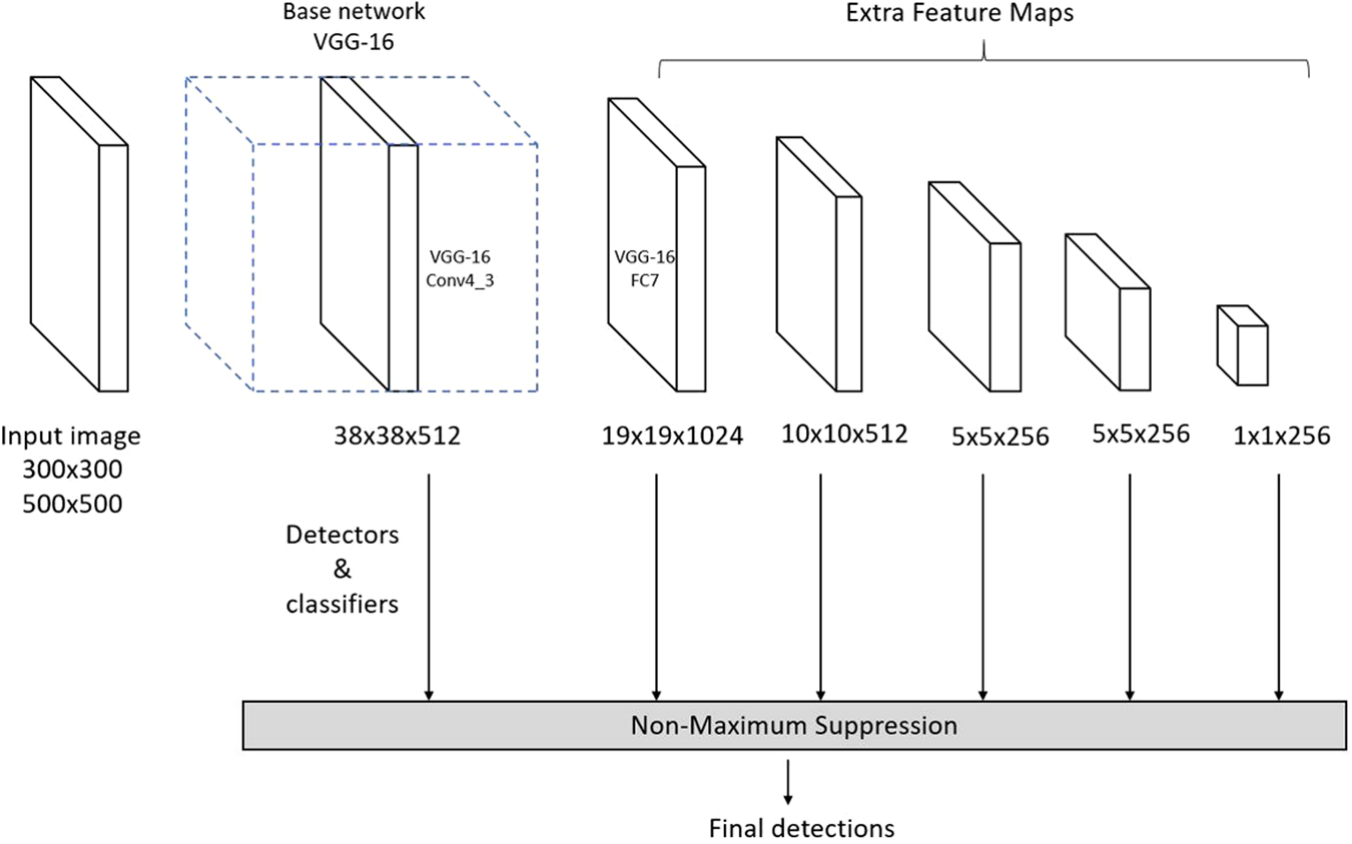
Architecture of Single shot multibox detector.

The label of SSD includes the class and the offsets from the default boxes. The default boxes are matched with ground truth if their intersection over union (IOU) is over 0.5. IOU is defined as *Area of Overlap*/*Area of Union*. The loss function of SSD combines a softmax loss for the confidence loss and a Smooth L1 loss for localization loss. The overall objective loss function is

$${L}_{tot}=\frac{1}{N}({L}_{conf}+\alpha {L}_{loc})$$

where N is the number of matched default boxes and *α* is set to 1 by cross-validation. The SSD framework is shown in Fig. 3. For more details of the SSD network and loss function please refer to^35^.

#### Training and Testing

Expert judges in VFSS image measurements manually annotated the hyoid bone location (coordinate of left corner, height and width) in each frame of the videos. To evaluate the reliability of the swallowing analysis, 10 swallow cases were utilized. Three experts analyzed the same 10 swallows. Inter-rater reliability was tested between raters and experts analyzed the same cases one month later for intra-rater reliability. ICC score were over 0.9 for all measures of reliability. The swallow data were split and distributed to each of the experts. Their annotations were considered as ground truth (gold standard). The data were randomly separated by patients: 70% of the patients were split into training data which contained around 30,000 frames with annotations, while 30% of the patients were split into test data which contained around 18,000 frames. We chose both VGG-16 and ResNet-101 as base networks, and considered two image resolutions for inputs: 300 × 300 and 500 × 500. We compared models trained on both base networks and both resolutions inputs. The input with size 500 × 500 should provide better performance as more details can be detected in higher resolution images. However, a larger image size increases the computation complexity. Furthermore, we compared the results with YOLO and Faster-RCNN and used a training procedure similar to the original papers. We chose 0.0005 as our learning rate with multi-steps, dividing by 10 for iteration 4000 and 8000. The momentum is 0.9 and gamma is 0.1 for the optimizers.

#### Evaluation of Accuracy

The performance of the detection module is measured by mean average precision (mAP), which is the most commonly used evaluation method for object detection. Average precision estimated whether detected bounding boxes match the corresponding ground truth. Mean average precision is the area below the precision-recall curve, which integrates precision and recall while varying from 0 to 1. As we have just one class to classify, mean average precision is the average precision for the hyoid bone class. The bounding box is labeled as true positive if IOU is greater than 0.5. Precision evaluates the fraction of true positive bounding box over all predictions and recall evaluates the fraction of the true positive detected bounding boxes among all ground truths.

## Results

**Figure 4:**
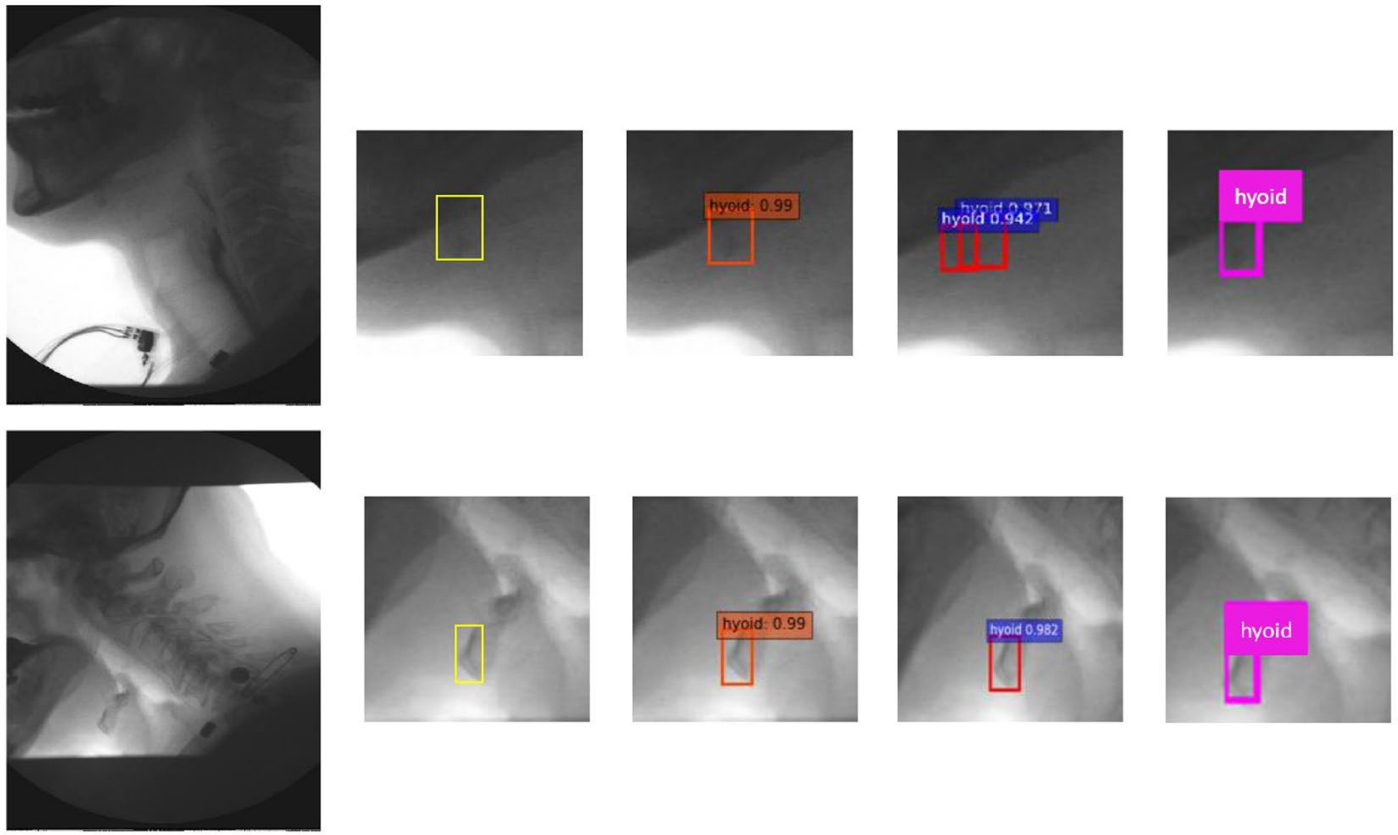
The identification of hyoid bone using different method: ground truth (yellow), SSD500-VGG (orange), Faster-RCNN (red), and YOLOv2 (pink).

Table 1 shows results of the state-of-the-art published methods on our VFSS image dataset. Overall, SSD method outperforms the results produced by YOLOv2 and Faster-RCNN. Among SSD method, VGGNet with input size of 500 × 500 produced the best result compared to ResNet and input size of 300 × 300. The mAP of SSD500-VGGNet is 89.14%, which is 0.11% better than using ResNet-101 as base network and 2.45% better than using the smaller image input size. Figure 3 shows the example results by manual segmentation, SSD500-VGGNet, Faster-RCNN and YOLOv2. Figure 4 illustrated the performance of these methods on VFSS images. We selected two different cases as examples: patient swallowing the bolus in neutral head position or in chin down position. In comparing automated hyoid detection to the ground truth, we used the bounding box to locate the hyoid bone. Most of the object detection methods use the bounding box to locate and classify the content inside. In the example case, all three tested methods revealed a positive result, detecting the hyoid bone location successfully. However, the Faster-RCNN method produced two regions of interest that it considered as the hyoid bone with a close confidence score.

**Table 1:** Comparison of mAP with different models.

Model	Mean average precision
YOLOv2	33.10%
Faster-RCNN + ZF	69.01%
SSD300-VGG	84.37%
SSD300-ResNet	81.92%
SSD500-VGG	89.14%
SSD500-ResNet	89.03%

**Figure 5:**
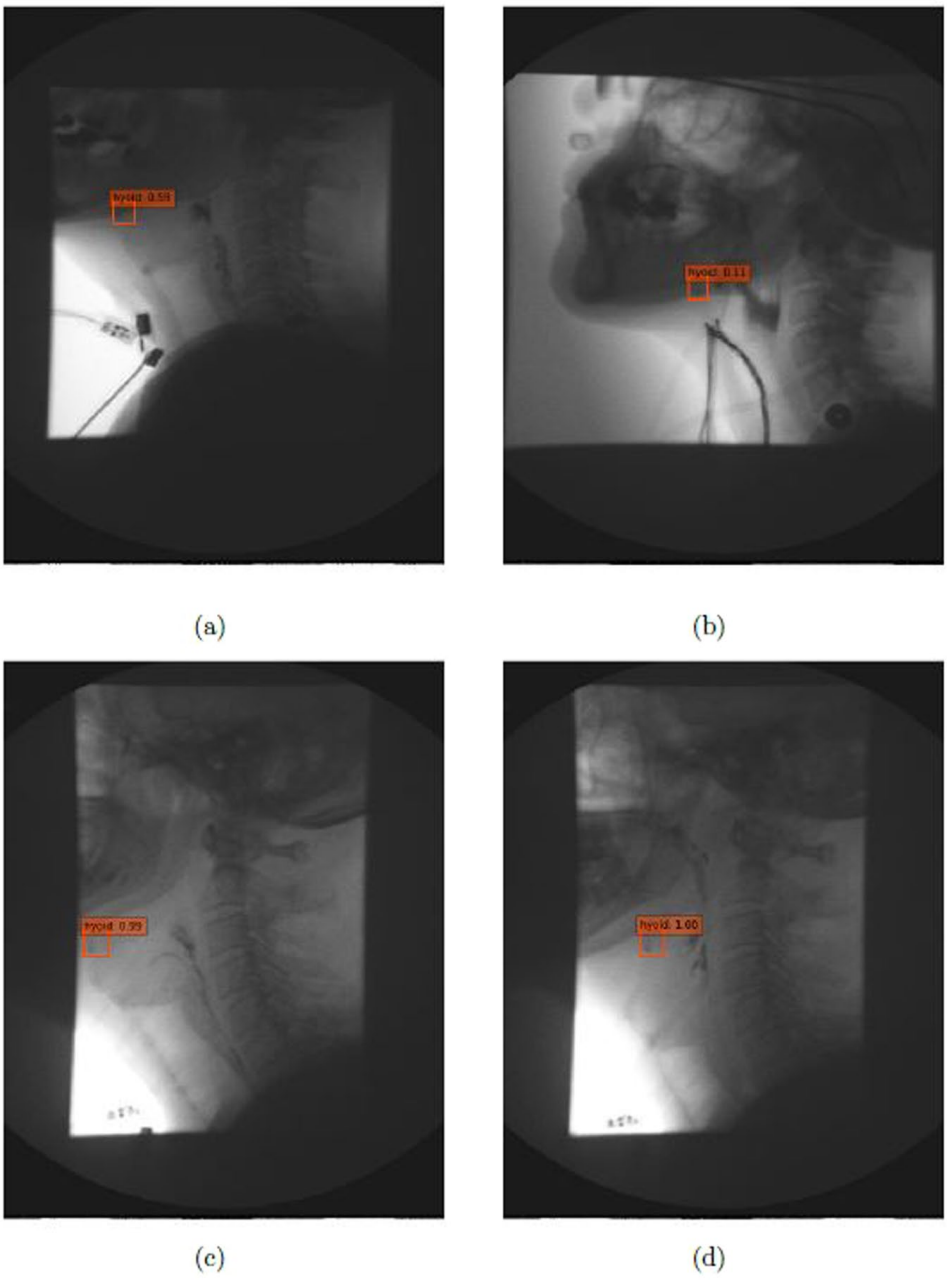
Results on different image conditions using SSD500-VGGNet: (**a**,**b**) hyoid bone hides behind mandible (**c**,**d**) hyoid bone is slightly blurred during motion.

Figure 5 illustrates results using the SSD500-VGGNet method with different hyoid bone locations (under the mandible and behind the mandible), and the results are shown with different image qualities. From these results, SSD500-VGGNet showed stable detection results, clearly finding the hyoid bone. When the hyoid bone is hidden behind the mandible in case (a) and (b), the algorithm detected the hyoid bone with a relatively low confidence score. It performed well in case (c) and (d) where the hyoid bone is present under the mandible.

**Figure 6:**
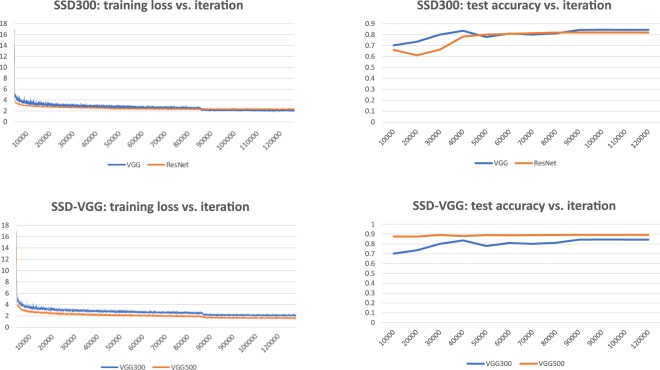
The influence of training loss and model performance of SSD models with different input sizes and pre-trained models.

Figure 6 shows the change of training loss function and the performance on test data during the training of SSD models. These figures illustrate how the performance of the model changes during training. The loss function dramatically decreased in the first 1000 iterations and the loss function only slightly decreased in the following training iterations. The training errors of SSD300-VGG were always higher than those of SSD500-VGG. SSD300 with different pre-trained models showed a similar training loss trend and test accuracy.

## Discussion

In this investigation, we aimed to detect the location of the hyoid bone in the videofluoroscopic images without human intervention. The hyoid bone is an important structure considered in dysphagia assessment. Its motion can be related to the severity of dysphagia and is used to assess treatment effectiveness. Manual tracking of hyoid bone data from VFSS is the gold standard accepted by experts and clinicians. Manually segmenting and annotating is time-consuming and prone to judgment error. The hyoid bone motion data presented in this paper can be applied in further investigations such as statistical methods and classification based on machine learning. A quantitative and qualified computer-aided system is highly desirable in clinical work in which the availability of an expert clinician to judge VFSS is not ubiquitous. Currently in dysphagia research, human judgment is necessary to annotate hyoid position in initial video frames. Elimination or mitigation of human judgment regarding hyoid motion could speed up image processing without compromising accuracy. The following sections discuss the performance of each method and possible factors that may have influenced the results.

We examined the performance of different object detection methods (Faster-RCNN, YOLOv2, and SSD) to locate hyoid bone in our VFSS image dataset. For the deep architecture, we employed the medium-size network VGGNet, the relatively larger-size network ResNet 101 for the SSD and a small network ZFNet for Faster-RCNN. YOLOv2 is from the original Darknet model^34^. The SSD500-VGGNet achieved better results than other CNN based models, indicating that it is the most suitable method for hyoid bone detection in VFSS images. It is not surprising that YOLO achieve the worst performance on VFSS data. The hyoid bone is a small object in the VFSS images. YOLOv2 is a fast object detection method but is weak for small object detection as it applies global features which doesn’t obtain enough details for small objects. SSD500 is better than SSD 300 in all settings by using ResNet-101 or VGGNet-16. The reasons might be as follows. SSD resizes the input images to a fixed size: SSD300 resizes the images into 300 × 300 while SSD500 resizes images into 500 × 500. The training errors of SSD300 model is higher than those in SSD500. Resizing the already small hyoid bone in images into a smaller size may result in a loss information. SSD300 cannot learn the details of the hyoid bone, which leads to worse performance. Furthermore, ResNet reached a similar mAP to VGGNet in SSD500 but it has worse performance in SSD300. ResNet-101 is a neural network with 101 layers, while VGG-16 has 16 layers. The similar performance in SSD500 may indicate that both networks provide detailed information for the added features layers. In the case of SSD300, the models with VGG networks had slightly smaller training loss after iteration 8000, which might explain why VGG performed better on test data. The SSD method is a powerful tool to detect the hyoid bone location, however, training SSD models with ResNet-101 and VGGNet with larger input size is time-consuming. We implemented our algorithms on the NVIDIA Tesla M40 GPU. It took over one week to train both the SSD500-VGG16 models and SSD500 with ResNet-101. The Faster-RCNN took only one day because ZFNet is a small neural network.

The hyoid bone moves upward and forward during a patient’s swallow. It will sometimes rise into the radiographic shadow of the mandible, obscuring its visibility by the judge/examiner. The judges must compare adjacent frames to infer the hyoid’s actual location when it is obscured by the mandible. Figure 5(a) and (b) show the detection of the hyoid bone. Although the confidence score is low, our algorithm can be considered successful because experts may not be able to locate the hyoid bone. Figure 5(c) and (d) are examples of blurred hyoid bone. The hyoid bone may be blurred when it moves quickly between two frames, but the algorithm can detect the moving hyoid bone with a high confidence score.

**Figure 7:**
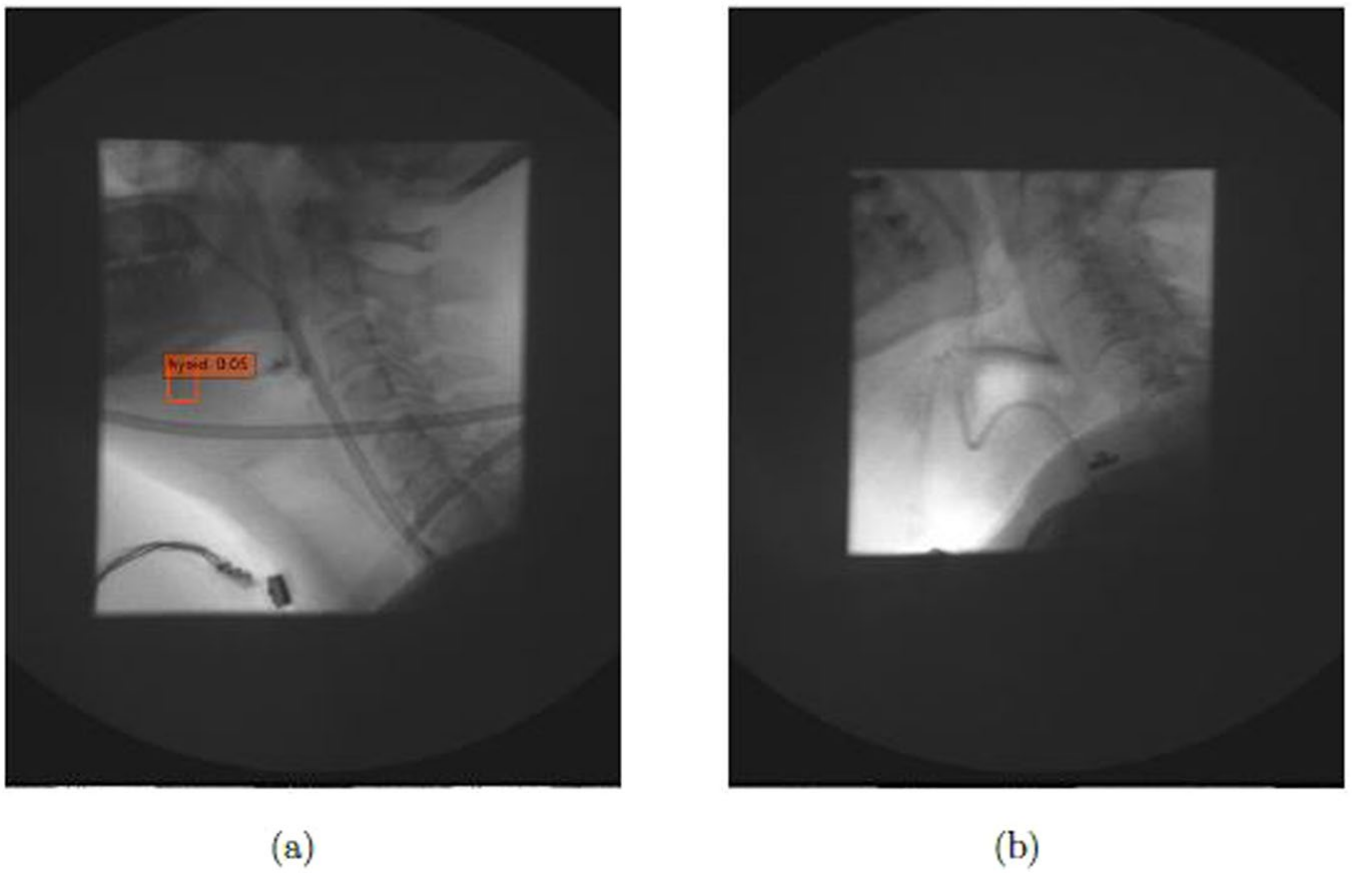
The cases which algorithm didn’t detect the hyoid bone (**a**) the case with low confidence score (**b**) the case totally not detected.

X-ray images vary in quality because clinicians control dosage to patients to the least amount of radiation as possible. Thus, as shown in the Fig. 5, the brightness and, contrast of each x-ray image is different, altering the amount of useful information in each image. As shown in Fig. 7, the SSD method detects the obscured hyoid bone location with a low confidence score or does not detect the hyoid bone location, similar to a guess when humans attempt to locate these cases. We know the location of the hyoid bone as the pre-knowledge, and seek to find a target around the predicted location while eliminating impossible regions one by one. The object detection algorithm classifies the regions based on the default boxes, which is a direct way to make the decision and can’t fully make use of outside information.

We investigated the performance of our model in the hyoid bone location task, however, our research had several limitations. X-rays images are often low quality, and the quality may vary from machine to machine. Whether the model can achieve similar performances across varied image quality requires further investigation. Furthermore, our investigation included data from 265 patients from the same hospital, which may provide limited diagnostic variability in patients. Additional data should be collected to improve the performance and stability of our model. Prior research^44^ indicated that Faster-RCNN with inception ResNet v2 has the best object detection results when compared to other modern object detection methods. Furthermore, several studies focused on small object detection, such as feature pyramid network^45^, which may be a direction for further research to increase the detection performance of the hyoid bone. For clinical relevance, future work should investigate automatic segmentation of hyoid bone areas, examine data to determine whether or not hyoid displacement is disordered, and determine if hyoid motion is the biomechanical etiology of impaired airway closure or upper esophageal sphincter opening. Moreover, since SSD detection methods detected the hyoid bone, future investigations will explore detecting other key components in videofluoroscopy images. Given the millions of VFSS studies implemented, high-accuracy component detection can save experts considerable time during their diagnosis.

## Conclusion

In this paper, we investigated hyoid bone detection in videofluoroscopy images using a deep learning approach. We used 1434 swallows on VFSS videos as our dataset. The hyoid bone location was manually annotated in each frame of the videos. We considered each frame as the single sample and trained 70% of the frames using state-of-the-art object detection methods. The SSD-500 model tracked the location of the hyoid bone on each frame accurately. Ideally, hyoid bone motion information can be used for physiological analysis. We believe that this proposed model has the potential to improve the diagnosis assessment of dysphagia.
